# Quantitative monitoring of leaf area index in wheat of different plant types by integrating NDVI and Beer-Lambert law

**DOI:** 10.1038/s41598-020-57750-z

**Published:** 2020-01-22

**Authors:** Chang-Wei Tan, Peng-Peng Zhang, Xin-Xing Zhou, Zhi-Xiang Wang, Zi-Qiang Xu, Wei Mao, Wen-Xi Li, Zhong-Yang Huo, Wen-Shan Guo, Fei Yun

**Affiliations:** 1 Jiangsu Key Laboratory of Crop Genetics and Physiology/Jiangsu Co-Innovation Center for Modern Production Technology of Grain Crops/Joint International Research Laboratory of Agriculture and Agri-Product Safety of the Ministry of Education of China, Yangzhou University, Yangzhou, 225009 China; 2 Station of Land Protection of Yangzhou City, Yangzhou, 225009 China; 3 National Tobacco Cultivation and Physiology and Biochemistry Research Centre/Key Laboratory for Tobacco Cultivation of Tobacco Industry, Henan Agricultural University, Zhengzhou, 450002 China

**Keywords:** Natural variation in plants, Plant ecology, Agroecology

## Abstract

Normalized difference vegetation index (NDVI) is one of the most important vegetation indices in crop remote sensing. It features a simple, fast, and non-destructive method and has been widely used in remote monitoring of crop growing status. Beer-Lambert law is widely used in calculating crop leaf area index (LAI), however, it is time-consuming detection and low in output. Our objective was to improve the accuracy of monitoring LAI through remote sensing by integrating NDVI and Beer-Lambert law. In this study, the Beer-Lambert law was firstly modified to construct a monitoring model with NDVI as the independent variable. Secondly, experimental data of wheat from different years and various plant types (erectophile, planophile and middle types) was used to validate the modified model. The results showed that at 130 DAS (days after sowing), the differences in NDVI, leaf area index (LAI) and extinction coefficient (k) of the three plant types with significantly different leaf orientation values (LOVs) reached the maximum. The NDVI of the planophile-type wheat reached saturation earlier than that of the middle and erectophile types. The undetermined parameters of the model (LAI = −ln (a_1_ × NDVI + b_1_)/(a_2_ × NDVI + b_2_)) were related to the plant type of wheat. For the erectophile-type cultivars (LOV ≥ 60°), the parameters for the modified model were, a_1_ = 0.306, a_2_ = −0.534, b_1_ = −0.065, and b_2_ = 0.541. For the middle-type cultivars (30° < LOV < 60°), the parameters were, a_1_ = 0.392, a_2_ = −0.88_1_, b_1_ = 0.028, and b_2_ = 0.845. And for the planophile-type cultivars (LOV ≤ 30°), those parameters were, a_1_ = 0.596, a_2_ = −1.306, b_1_ = 0.014, and b_2_ = 1.130. Verification proved that the modified model based on integrating NDVI and Beer-Lambert law was better than Beer-Lambert law model only or NDVI-LAI direct model only. It was feasible to quantitatively monitor the LAI of different plant-type wheat by integrating NDVI and Beer-Lambert law, especially for erectophile-type wheat (R^2^ = 0.905, RMSE = 0.36, RE = 0.10). The monitoring model proposed in this study can accurately reflect the dynamic changes of plant canopy structure parameters, and provides a novel method for determining plant LAI.

## Introduction

The leaf area index (LAI), the leaf orientation value (LOV), and the extinction coefficient (k) are important structural parameters of crop populations. By affecting light distribution, they directly affect crop photosynthetic efficiency, and ultimately show an impact on crop biological yield and its distribution in various plant organs^1^.

Remote sensing technology could provide a practical method for crop LAI estimation, rather than a slow, expensive and complicated chemical method. The advantage of the remote-sensing method is that it can obtain plant canopy information on a large scale without disrupting the normal growth of plants^2,3^. Studies using remote sensing to monitor agronomic parameters have been extended from crop soils^4–6^, to fresh leaves^7–9^ and entire crop layers^10,11^. Previous studies have shown that a macronutrient (Nitrogen, Phosphorus, Potassium, Calcium and Magnesium) deficiency in wheat lowers chlorophyll concentrations, increases reflectivity in the visible range, and causes the red edges to shift towards the shorter or longer wavelengths^12^. Several studies have estimated water content, nitrogen and grain protein content using spectral data^13^. However, many factors are needed to be considered to study the reflectivity of crop canopy. These include the complexity of canopy characteristics, changes in leaf internal structure and soil background effects^14,15^. The complexity of canopy structure have played a decisive role in solar radiation interception, absorption and scattering. The results have shown that canopy structure mainly depends on leaf distribution and area. Leaf angular distribution is one of the key parameters to simulate the radiative transfer of vegetative canopy and the balance of energy and mass^16^. Traditionally, the concept of LAI have been used in agriculture and ecology to measure the development and yield of crops, to compare among them and to schedule irrigation and amendments during the crop development^17^. While exploring k, variability in k values is not related to fraction of light intercepted, time of day, or incident solar radiation.

The LOV is an index indicating the degree of leaf blades point upward or downward. The bigger the LOV, the closer the leaf blades pointing upward to vertical, and the plant is erectophile. The smaller the LOV, the deeper the blades pointing downward, and the plant is flat. Regarding the study of light distribution in crop populations, Monsi and Saeki has expanded the Beer-Lambert law into plant populations and proposed the exponential relationship of light distribution in plant populations^18^: F = −ln(I/I_0_)/k, where F is the LAI, I_0_ and I represents the light intensity above and below the canopy respectively, and k is the extinction coefficient. Direct measurements and long-term monitoring of LAI are difficult, especially in crops and forests, but indirect methods are suitable for long-term continuous monitoring^19^. In many researches, the extinction coefficient is usually expressed as a single factor function of solar elevation Angle or leaf area index. To simplify calculations, many studies have monitored LAI with a constant k over the growing period^20,21^. Subsequently, numerous scholars have carried out various researches and discussions on this law. Some scholars use the Beer-Lambert extinction law to estimate the extinction coefficient from solar elevation to reveal the expected LAI dynamics^22^. Although the geometric distribution of leaf area has a potential impact on a plant’s ability to intercept light, but the previous equation do not contain any term to account for it. Some research has shown that the contribution of plant structure complexity to the law is evaluated by quantifying the geometric distribution of leaf area by the fractal dimension of leafless plant structure^23^.

Although the above achievements have boosted the physiological research of crop cultivation, the acquisition of morphological parameters is time-consuming, labor-intensive and destructive, so it is difficult to use in guiding cultivation practice. In recent years, spectral vegetation indices (such as normalized difference vegetation index (NDVI)) have been widely used to evaluate the fractional vegetation cover qualitatively and quantitatively and monitor crop growth. The NDVI is well correlated with LAI and is more sensitive to changes in the crop canopy when the LAI is low (during the early stage), with the signal becoming saturated when the crop canopy closes^24,25^. Through NDVI from spectroradiometer and its slope curves to monitor rice development, the results demonstrate that they could be used as cultivar-independent phenological indicators^26^. However, using NDVI to conduct research on light distribution in crop populations is still barely reported.

The objectives of this study were to develop a more accurate LAI monitoring model, and to identify the feasibility of monitoring wheat LAI by integrating remote sensing variables and Beer-Lambert law. Our study provided a basis for fast and non-destructive identification of light distribution characteristics of wheat with different plant-type using the hyperspectral remote sensing technology.

## Results

### Changes in NDVI, LAI and k of various plant types at different growth stages

**Figure 1:**
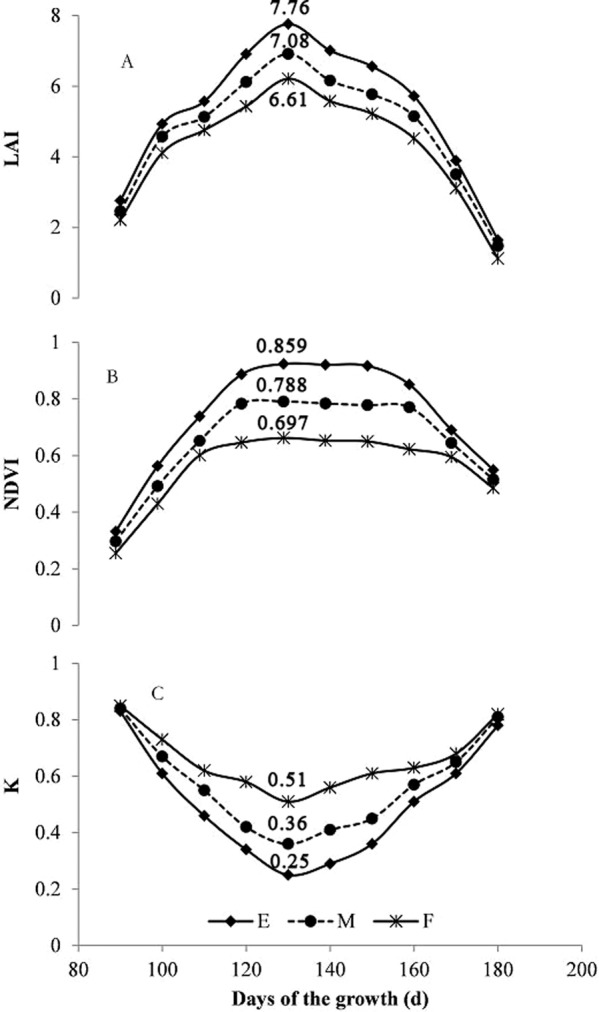
Dynamic curve of NDVI, LAI and k. E: Erectophile-type, M: Middle-type, F: Planophile-type (the same as below).

The changes in NDVI, LAI and k of different plant-types throughout the growth stages has been presented in Fig. 1. The LOVs calculated from the corresponding measured canopy structure data were 78.8°, 48.6°, and 25.4°, for erectophile, middle and planophile types, respectively.

As shown in Fig. 1A, the LAI exhibited single peak curves along the growth process. From about 90 to 120 DAS (days after sowing), LAI values were increasing, without significant differences among the three wheat cultivars. From 120 to 130 DAS, the LAI kept increasing as the plants grew vigorously, and each cultivar clearly demonstrated its own plant type related characteristics. With the increase of LOV value, the more compact the plant, the higher the production density and LAI. Hence, the LAI differences among the three cultivars increased, reaching the maximum at 130 DAS. Then the LAI differences gradually decreased. Presumably, the rate of photosynthesis reduces at this stage due to senescence and withering of lower layer leaves. These resulted in smaller LAI, hence smaller differences.

Figure 1B demonstrates the first increased and then decreased single peak changes of NDVI of three cultivars and their NDVI differences, along with the changes of LAI. From about 90 to 120 DAS, along with the increase of plant height, the number of leaves, and the single leaf area, the LAI increased accordingly. Then the absorbed red light increased, resulting in lowered intensity of the red-light reflectance, and increased NDVI values. Generally, it was thought that the NDVI value was primary affected by leaf pigment and LAI. Nevertheless, the NDVI values differed only slightly at this stage. The primarily reason was that the plants were still short then, the plant type related characteristics hadn’t been fully exhibited yet. Between 120–130 DAS, the changes in NDVI values of the three cultivars gradually increased, reaching a maximum at 130 DAS. The NDVI values and their differences gradually decreased afterwards and became very weak after 160 DAS, presumably due to the differences in LAI generated by the planting density. This was consistent with the conclusion reached by Suryanarayana *et al*. on the wheat NDVI value study^26^. In addition, the NDVI value increased with the increase of LOV from plant types of planophile to erectophile. The NDVI value reached saturation at around 110 DAS for the planophile type, followed by the middle type at 120 DAS, and finally the erectophile type at 130 DAS. Therefore, it indicated that the saturation of NDVI was related to the plant type, earliest for the planophile type, then the middle type, and latest for the erectophile type. Compared to the middle and erectophile types, the planophile-type leaves spread earlier and faster, and the coverage was higher in the early stage, which greatly affected the reflection spectrum.

According to Beer-Lambert law, when LAI was a constant, the bigger the k value, the smaller the light energy ratio (I/I_0_) through the canopy. While holding I/I_0_ constant, the LAI was inversely proportional to the k value, and k reflects the degree of light reduction within the canopy, which could be taken as the amount of light interception per unit leaf area. Besides LAI, k was also related to the angle between the blade and the horizontal plane. As shown in Fig. 1C, the smaller the LOV value, the larger the k value. The k value demonstrated a concave curve along with the growth process, first decreasing and then increasing. From about 90 to 120 DAS, the LAI values were increasing, the leaf photosynthetic capacities were rising, leading to the k values reduced at varying degrees. Nevertheless, the differences in k values of the three plant types were small, when reaching maximum at 130 DAS, with the lowest k value for the planophile type. Later, the k values increased to various extents. In addition, it was also found that along with the growth, change of k value in the same variety was greater than that between varieties of different plant types. The plausible explanation was that along with the growth process, the number of leaves increased, the area of single leaves increased, and the planting density of various plant types varied.

### Relation analysis between NDVI and LAI for various plant types

**Figure 2:**
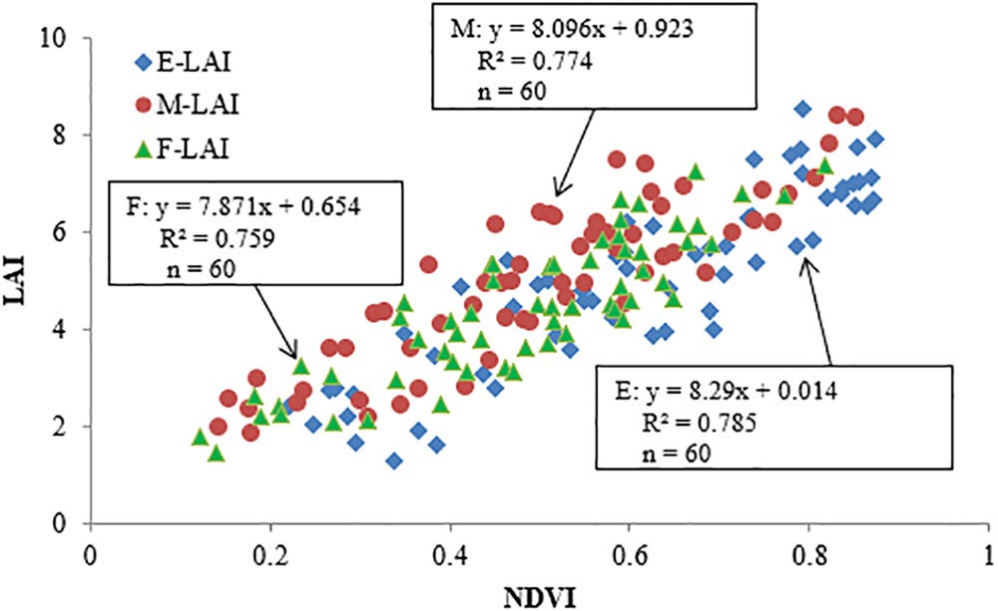
NDVI-LAI direct models under various plant types.

As shown in Fig. 2, the correlations between NDVI and LAI were extremely significant for three plant types, with coefficients of determination (R^2^) values of erectophile, middle and planophile types being 0.785, 0.774 and 0.759, respectively. To monitor wheat LAI directly, NDVI was selected as an independent variable, LAI as a dependent variable, and the NDVI-LAI direct models of erectophile, middle and planophile types were constructed respectively (Fig. 2). Furthermore, it was observed that the NDVI can be used to in directly monitor erectophile-type, middle-type and planophile-type wheat LAI.

### Correlation analysis of NDVI with I/I_0_ and k for various plant types

**Figure 3:**
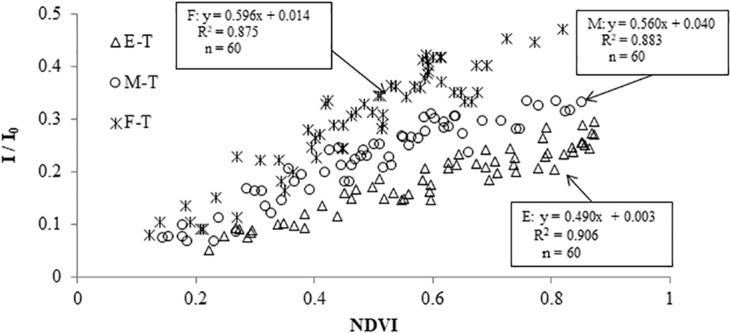
Relationships between I/I_0_ and NDVI under various plant types (T represented I/I_0_).

**Figure 4:**
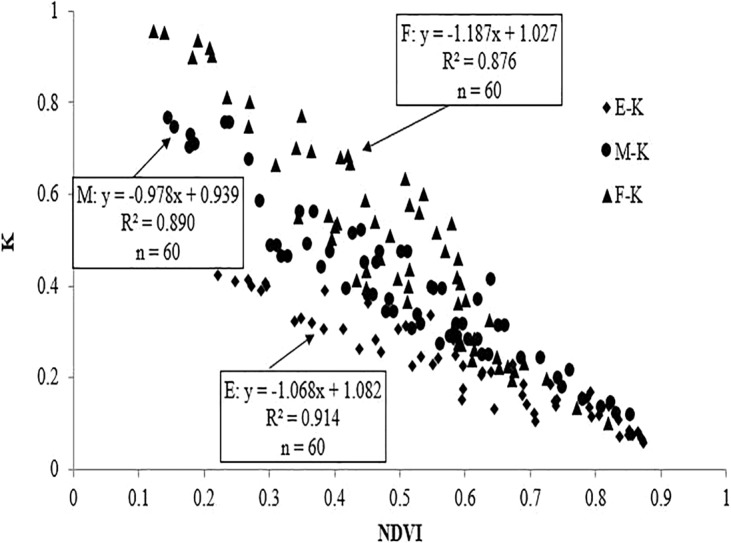
Relationships between k and NDVI under various plant types.

To more accurately monitor the LAI change in wheat with combined remote sensing technology and Beer-Lambert law, the wheat NDVI of various plant types at 130 DAS were subjected to statistical correlation analysis with I/I_0_ (Fig. 3) and k values (Fig. 4), respectively. The results demonstrated that the correlation of NDVI with k, and with I/I_0_ for the three plant types was extremely significant, with R^2^ of both above 0.87. Regarding the linear correlation between I/I_0_ and NDVI, the erectophile type showed the strongest correlation (R^2^ = 0.906, P < 0.01), followed by the middle type (R^2^ = 0.883, P < 0.01), and the planophile type (R^2^ = 0.875, P < 0.01). With respect to the linear correlation between k and NDVI, it was similar to the abovementioned trend, that is, the erectophile type had the highest correlation (R^2^ = 0.914, P < 0.01), followed by the middle type (R^2^ = 0.890, P < 0.01), and planophile type (R^2^ = 0.876, P < 0.01). Considering all three plant types, NDVI was linearly correlated with I/I_0_ and k. While the correlation was different for different LOVs, that is, various plant types. Therefore, the regression model of I/I_0_ and k using NDVI as the independent variable for the three plant types was established respectively as:

1 $$I/{I}_{0}={a}_{1}\times NDVI+{b}_{1}$$

2 $$K={a}_{2}\times NDVI+{b}_{2}$$

3 $$LAI=-\,{ln}(\frac{I}{{I}_{0}})\times \frac{1}{K}$$

When substitute the Eq. (3) with Eqs. (1) and (2) the Beer-Lambert law could be transformed into:

4 $$LAI=\frac{-ln({a}_{1}\times NDVI+{b}_{1})}{{a}_{2}\times NDVI+{b}_{2}}$$

where a_1_, a_2_, b_1_, and b_2_ are the undetermined parameters.

According to the results of Figs. 3 and 4, the undetermined parameters (a_1_, a_2_, b_1_, and b_2_) in Eq. (4) are calculated and listed in Table 1.

**Table 1:** Monitoring model in various ranges of LOV.

LOV	Eq. (4): ${\boldsymbol{L}}{\boldsymbol{A}}{\boldsymbol{I}}{\boldsymbol{=}}\frac{{\boldsymbol{-}}{\boldsymbol{\ell }}{\boldsymbol{n}}({{\boldsymbol{a}}}_{{\bf{1}}}{\boldsymbol{\times }}{\boldsymbol{N}}{\boldsymbol{D}}{\boldsymbol{V}}{\boldsymbol{I}}{\boldsymbol{+}}{{\boldsymbol{b}}}_{{\bf{1}}})}{{{\boldsymbol{a}}}_{{\bf{2}}}{\boldsymbol{\times }}{\boldsymbol{N}}{\boldsymbol{D}}{\boldsymbol{V}}{\boldsymbol{I}}{\boldsymbol{+}}{{\boldsymbol{b}}}_{{\bf{2}}}}$			
	a_1_	b_1_	a_2_	b_2_
LOV ≥ 60°	0.306	−0.065	−0.534	0.541
30° <LOV <60°	0.392	0.028	−0.881	0.845
LOV ≤ 30°	0.596	0.014	−1.306	1.130

### Model validation

**Figure 5:**
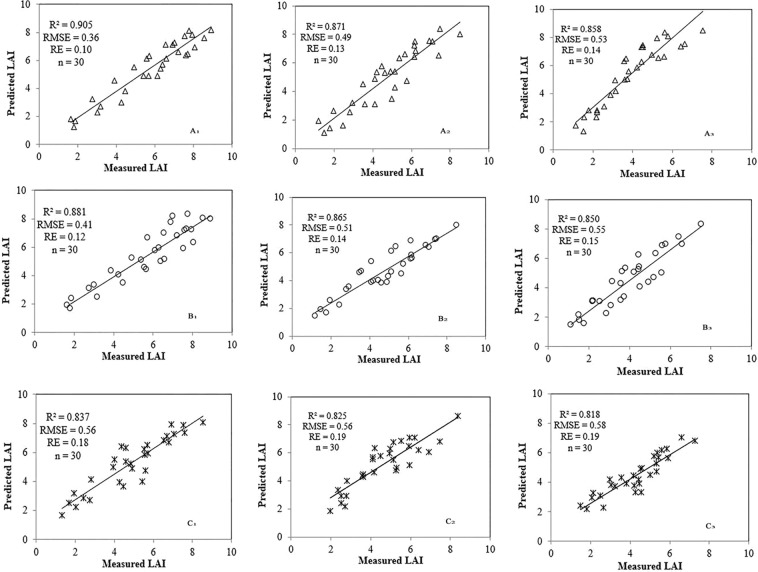
Evaluating the monitoring models of erectophile-type (A_1_, B_1_, C_1_), middle-type (A_2_, B_2_, C_2_) and planophile-type (A_3_, B_3_, C_3_) wheat LAI. A_1_, A_2_, A_3_ represented LAI deduced by Eq. (4), respectively. B_1_, B_2_, B_3_ represented LAI deduced by Eq. (3), respectively. C_1_, C_2_, C_3_ represented LAI deduced by the NDVI-LAI direct model, respectively.

In order to test the reliability of the model, the measurement data of 2018, which was an experiment repeat of 2017, was used in the validation. The experiment field was divided into three sections (the erectophile type LOV ≥ 60°, the middle type 30° < LOV < 60° and the planophile type LOV ≤ 30°). The measured LAI and the calculated NDVI based on ground hyperspectral data were used to validate Eq. (4) for the three LOV ranges, and the values were also compared to the corresponding prediction values from Eq. (3) and the NDVI-LAI direct models, as shown in Fig. 5. The measured LOV values were basically in [20°, 30°], [40°, 50°] and [70°, 80°] for the planophile, middle and erectophile types, respectively. Based on the analysis from Table 2, it was shown that the measured and the simulated LAI values were significantly correlated for the three models, with all the R^2^ above 0.80. R^2^ of Eqs. (4) and (3) were obviously higher than R^2^ of the NDVI-LAI direct models, and their root mean square error (RMSE) and relative error (RE) were obviously lower than RMSE and RE of the NDVI-LAI direct models, which indicated that the Eqs. (4) and (3) were more suitable for quantitatively monitoring wheat LAI than the NDVI-LAI direct models. Else, when LOV ≤ 30°, the prediction results of Eqs. (4) and (3) were close, and the prediction of Eq. (4) was valid. When LOV ≥ 60°, the determination coefficient (R^2^ = 0.905, P < 0.01) obtained from Eq. (4) was the biggest regarding the erectophile type, with a relatively small RMSE and RE of 0.36 and 0.1, respectively. Therefore, it was indicated that it is possible to predict the dynamic changes of wheat canopy structure using the Beer-Lambert law with NDVI obtained by hyperspectral technique, and it was highly accurate.

**Table 2:** Results of evaluating the LAI monitoring models.

LOV	Eq. (4): ${\boldsymbol{L}}{\boldsymbol{A}}{\boldsymbol{I}}{\boldsymbol{=}}\frac{{\boldsymbol{-}}{\boldsymbol{\ell }}{\boldsymbol{n}}({{\boldsymbol{a}}}_{{\bf{1}}}{\boldsymbol{\times }}{\boldsymbol{N}}{\boldsymbol{D}}{\boldsymbol{V}}{\boldsymbol{I}}{\boldsymbol{+}}{{\boldsymbol{b}}}_{{\bf{1}}})}{{{\boldsymbol{a}}}_{{\bf{2}}}{\boldsymbol{\times }}{\boldsymbol{N}}{\boldsymbol{D}}{\boldsymbol{V}}{\boldsymbol{I}}{\boldsymbol{+}}{{\boldsymbol{b}}}_{{\bf{2}}}}$			Eq. (3): ${\boldsymbol{L}}{\boldsymbol{A}}{\boldsymbol{I}}{\boldsymbol{=}}{\boldsymbol{-}}{\boldsymbol{\ell }}{\boldsymbol{n}}(\frac{{\boldsymbol{I}}}{{{\boldsymbol{I}}}_{{\bf{0}}}}){\boldsymbol{\times }}\frac{{\bf{1}}}{{\boldsymbol{K}}}$			NDVI-LAI direct model		
	R^2^	RMSE	RE	R^2^	RMSE	RE	R^2^	RMSE	RE
LOV ≥ 60°	0.905**	0.36	0.10	0.881**	0.41	0.12	0.837**	0.56	0.18
30° <LOV <60°	0.871**	0.49	0.13	0.865**	0.51	0.14	0.825**	0.56	0.19
LOV ≤ 30°	0.858**	0.53	0.14	0.850**	0.55	0.15	0.818**	0.58	0.19

^**^Indicated significant at 0.01 level.

## Discussion

LOV was one of the important parameters to simulate canopy radiation transfer and mass balance. The quantitative study of Beer-Lambert law was carried out by remote sensing and measured canopy structure parameters of wheat cultivars with different LOVs. Previous work has suggested that LOV could be measured to check effectiveness of visual ratings of leaf orientation^27^. This paper analyzes the potential of NDVI to quantitatively modify the Beer-Lambert law to quantify wheat leaf area index. The results of this study were tested with data from a different year to obtain the modified equation of Beer-Lambert law using spectral remote sensing technology in different LOV ranges.

NDVI is one of the most important vegetation indices in vegetation remote sensing^24,26^. It has a close relationship with plant parameters such as crop LAI^28^. It had been widely used in remote sensing monitoring of crop growth^29^. This is the basis for selecting NDVI to monitor different plant-type wheat LAI by integrating Beer-Lambert law in this study. The NDVI-LAI direct model for quantitatively monitoring wheat LAI is simple and feasible, but its accuracy was significantly lower than that of Eqs. (4) and (3). So far, there are a few studies on quantitatively monitoring of different plant-type wheat LAI by integrating NDVI and Beer-Lambert law^30^. Therefore, the quantitative monitoring of different plant-type wheat LAI by integrating NDVI and Beer-Lambert law had good application and innovation.

Structure of crops has a certain influence on the directional reflectivity of canopy^31^. Some researches put forward the remote monitoring model based on vegetation index, realizing the identification of various plant types^32^. In this study, the larger the LOV, the more erectophile the plant type, the stronger the monitoring ability of the above model (Eq. (4)) to the wheat LAI. In recent years, as the wheat breeding favored the erectophile-type cultivars, the results of this study were valuable in actual wheat production.

The present paper has only studied the light intensity above the canopy and above the base, without exploring the vertical distribution of structural parameters along various layers of the canopy. This is a limitation of the study. Moreover, spectral or image information from most optical sensors is obtained in the direction of the vertical canopy of the plant^32^. It could be predicted that in the near future, there would be more sensors to meet the requirements of different parameters, Therefore, whether the modified Beer-Lambert law is applicable to the vertical distribution of studied structural parameters along various layers of the canopy needs to be verified. In this study, only one variety was selected for each plant type. The wheat varieties with different growth period were not selected as test subjects, and the climatic conditions in the test area were not much different. Whether it can be used as a general model, more varieties of verification are needed. This study provided a potential tool for quantitatively monitoring different plant-type wheat LAI and a basis for quantitative remote monitoring of crops.

## Conclusions

It was feasible to monitor different plant-type wheat LAI by integrating NDVI and Beer-Lambert law, and with a better accuracy. It showed a distinctive advantage of fast, simple, and destruction-free features compared to the original Beer-Lambert law, and had a higher capability for quantitatively monitoring wheat LAI than the NDVI-LAI direct model. In addition, it was found that the more erectophile the plant type, the stronger the monitoring ability of the method by integrating NDVI and Beer-Lambert law. The results were valuable in the practice for breeding and cultivation.

## Materials and Methods

### Experimental design

**Figure 6:**
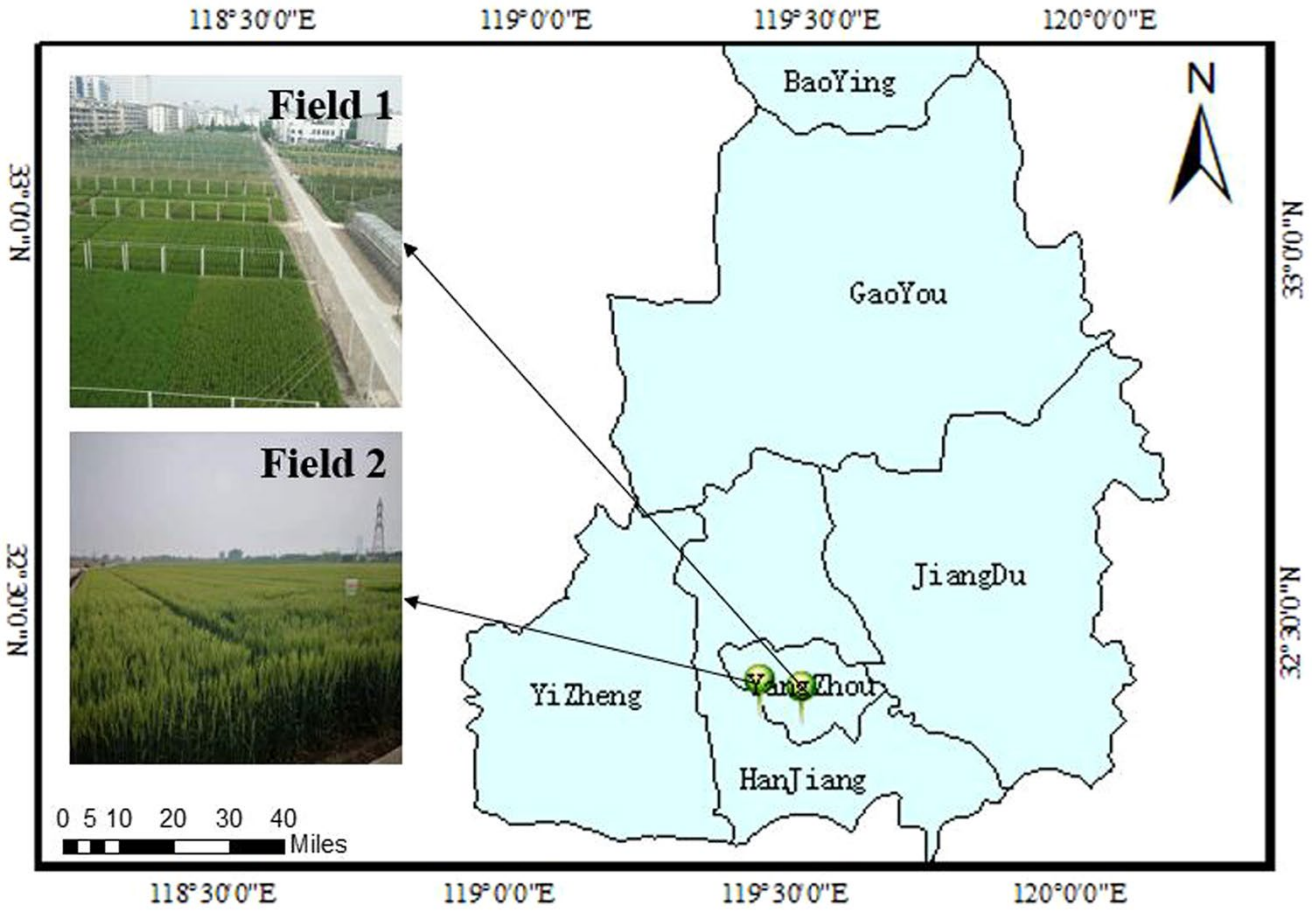
Experimental field location.

The experiments were conducted in No. 1 experimental field of Agricultural College of Yangzhou University from 2016 to 2017 (119°23′26″E, 32°23′53″N), and in the experimental farming of Yangzhou university in 2018 (119°26′22″E, 32°25′34″N) as shown in Fig. 6. A total of three representative wheat cultivars (lines) were selected including *Yumai 41*, *Zhengfeng 3* and *Jingdong 8*. Among them, *Yumai 41* was erectophile-type cultivar (line), *Zhengfeng 3* was middle-type cultivar (line), and *Jingdong 8* was planophile-type cultivar (line). The classification of wheat type was according to the method of Pepper *et al*.^33^. In brief, LOV ≥ 60° was an erectophile type, 30° < LOV < 60° was a middle type, and LOV ≤ 30° indicates a planophile type. The soil was loam textured. The nutrient content in 0–30 cm soil layer included organic matter 1.73–2.08%, ammonium nitrogen 10.4–12.8 mg·kg^−1^, nitrate nitrogen 15.82–17.76 mg·kg^−1^, available phosphorus 16.4–18.3 mg·kg^−1^, and available potassium 215–220 mg·kg^−1^. The area for each plot was 8 m × 8 m, with three replications. The planting density was the same as in the production field (Table 3). Training data consisted of 60 samples from 2016 and 2017, and test data comprised 30 samples from 2018.

**Table 3:** Plant type and basic seedlings.

Plant type	Basic seedlings (×10^4^ plants·ha^−1^)
Erectophile-type	450
Middle-type	375
Planophile-type	300

### Test items and test methods

#### Determination of spectral reflectance

The FieldSpec4 spectrometer from ASD (United States) was used to determine the spectral reflectance, with a viewing angle of 25° in the wavelength range of 350–2500 nm. There were 2,151 output bands after resampling. The measurements were performed on sunny cloudless and light breezy days. Starting from about 90 DAS, the spectral reflectance of plants in the representative plots was determined about every ten days, which were about Day 90, 100, 110, 120, 130, 140, 150, 160, 170 and 180 (In case of bad weather, the specific time could be determined according to the weather conditions.), between GMT 10:30–14:00. During the measurement, the probe was placed vertically downward at 60 cm from the top of the wheat canopy. For each plot, it was measured 20 times with uniformly changed positions within 1 m^2^. The average value was taken as the spectral reflectance of that plot. The reference correction was immediately conducted before and after the measurement for each plot. The following equation was used to calculate NDVI:

5 $$NDVI=\frac{{R}_{NIR}-{R}_{red}}{{R}_{NIR}+{R}_{red}}$$

where *R*_*NIR*_ and *R*_*red*_ are the average reflectance in the near infrared wavelength (760–900 nm) and the red-light wavelength (630–690 nm), respectively.

#### Determination of canopy structure parameters

k: The measurements were conducted on cloudy or overcast days. The handles with fisheye lenses were mounted in the center of each row and each column respectively, approximately 1 cm above the ground surface and leveled. The images were taken when there were no external interferences such as nobody was shown on the computer display. After computer image digitization processing and analysis with special software (CI-110), k values could be obtained.

LOV: One representative plant from each plot was selected. The length of the leaf (L, cm), the distance from leaf base to the flagging point of the measured leaf (h, cm), and the angle between the leaf and the stem (*a*, °) were measured with rulers and protractors, respectively. The LOV was calculated according to the following equation:

6 $$LOV=\mathop{\sum }\limits_{i=1}^{n}[(90^\circ -a){(h/L)}_{i}/n]$$

where *a* is the angle between the leaf and the stem, *h* is distance from leaf base to the flagging point of the leaf, *L* is the leaf length, and *n* is the number of measured leaves.

Light intensity (I): I was measured above the canopy and above the base using a LI-191SA radiation sensor for the sampling point, respectively.

LAI: The dry weight method was adopted, that was, after the areas of 5–10 leaves from the same treatment were measured, and the remaining dry weight of the leaves was measured. The average specific leaf weight was calculated, and then the leaf area of the sample was calculated, and then corrected by a CI-203 laser leaf area meter.

Beer-Lambert law:

7 $$I={I}_{0}\times {e}^{-KF}$$

Equation (7) was transformed to:

8 $$F=-ln(\frac{I}{{I}_{0}})\times \frac{1}{K}$$

where F was the LAI, I_0_ and I are the light intensities above the canopy and above the base, respectively, k is the canopy extinction coefficient, and I/I_0_ is the light transmittance.

#### Model establishment and validation

The light distribution parameters and NDVI data were input into the computer. The variance and correlation analysis were performed with SAS 6.10 statistical software. Further, the root mean square error (RMSE) and relative error (RE) calculated by the following equation were used to validate the model. Higher the value of R^2^, lower would be RMSE and RE values and higher would be the accuracy of quantitatively monitoring LAI with the NDVI-based model. Equations (9) and (10) were used to calculate RMSE and RE, respectively:

9 $$RMSE=\sqrt{\frac{1}{n}\mathop{\sum }\limits_{i=1}^{n}{({y}_{i}-{\hat{y}}_{i})}^{2}}$$

10 $${\rm{RE}}=({y}_{i}-{\hat{y}}_{i}){/{\rm{y}}}_{{\rm{i}}}\times 100$$

where ${{\rm{y}}}_{{\rm{i}}}$ and ${\hat{{\rm{y}}}}_{{\rm{i}}}$ represent measured and predicted LAI, respectively, and *n* is the number of samples.
